# Postoperative complications after paediatric cardiac surgery: the role of ethnicity and deprivation – a national cohort study

**DOI:** 10.1136/archdischild-2025-329267

**Published:** 2025-12-13

**Authors:** Hannah K Mitchell, Meriam Abdelmoumene, Ferran Espuny-Pujol, Gareth Ambler, Julie Taylor, Rodney C G Franklin, Christina Pagel, Sonya Crowe, Katherine Brown

**Affiliations:** 1 UCL GOS Institute of Child Health, London, UK; 2 UCL Medical School, London, UK; 3 Department of Computer Science, University of Reading, Reading, UK; 4 University College London, London, UK; 5 Clinical Operational Research Unit, University College of London, London, UK; 6 Paediatric Cardiology Department, Royal Brompton and Harefield NHS Trust, London, UK; 7 Great Ormond Street Hospital NHS Biomedical Research Centre London and Institute of Cardiovascular Science, Great Ormond Street Hospital NHS Foundation Trust, London, UK

**Keywords:** Cardiology, Child Health, Epidemiology, Healthcare Disparities, Paediatrics

## Abstract

**Aim:**

To understand whether the risk of complications following paediatric cardiac surgery differs according to a child’s ethnicity or the degree of residential deprivation.

**Methods:**

We conducted a retrospective cohort study using data from the National Congenital Heart Disease Audit, including children younger than 18 years who underwent cardiac surgery between April 2015 and March 2022 across 10 paediatric cardiac surgical centres in England and Wales. We examined the occurrence of six defined postoperative complications and used previously reported descriptive models to account for case complexity. Multivariable analysis was used to assess the association between complications and individual ethnicity and socioeconomic deprivation.

**Results:**

There were 23 423 30-day postoperative episodes. Children of Asian ethnicity were more likely to have a functionally univentricular heart or congenital cardiac risk factors, while children of Black ethnicity were more likely to have Down syndrome and prematurity than children of the remaining ethnicities. Children from the most versus the least deprived areas had higher rates of congenital comorbidity, functionally univentricular heart, high illness severity and urgent operations. After adjusting for case complexity, children from high compared with low deprivation areas had greater odds of prolonged pleural effusion (p=0.05), extracorporeal life support (p=0.001) and unplanned reintervention within 30 days (p=0.04).

**Conclusions:**

Greater area deprivation in England is associated with increased preoperative medical complexity and a higher incidence of certain postoperative complications among children with congenital heart disease (CHD). Further research is needed to explore the relationship between ethnic background and perioperative outcomes in CHD and to develop pathways to improvement based on social factors.

**WHAT IS ALREADY KNOWN ON THIS TOPIC**

- Children with congenital heart disease (CHD) are more likely than their unaffected peers to live in deprived areas and to be of minority ethnic background. Children with CHD residing in addresses of high deprivation have higher infant mortality rates than those residing in low deprivation areas. Complications of heart surgery are important outcomes that are associated with higher mortality and poorer functional outcomes.

**WHAT THIS STUDY ADDS**

- This study shows that children residing in more deprived areas had greater preoperative case complexity. After adjusting for case complexity, deprivation was associated with a higher risk of three of the six defined complications: prolonged pleural effusion, unplanned reintervention and the need for extracorporeal life support. The relationship between ethnicity, case complexity and postoperative complications is unclear.

**HOW THIS STUDY MIGHT AFFECT RESEARCH, PRACTICE OR POLICY**

- Our study findings highlight the need to better understand how social and structural disadvantage influences surgical recovery. Identifying modifiable contributors to disparities in postoperative complication rates could inform improvements in perioperative care and in follow-up support for higher-risk children.

## Introduction

 Paediatric cardiac surgery 30-day mortality is <2%; therefore, attention has shifted towards quality of survivorship, which is influenced by postoperative complications.^1^,^3^ A UK study found 21.8% of cardiac procedures were followed by at least one significant complication,^2^ affecting length of stay and hospital costs^4 5^; psychosocial well-being^6^ and neurodevelopment.^7^

Studies have shown a higher incidence of congenital heart disease (CHD) in children of Asian and black ethnicity, compared with white children in the UK.^8^,^10^ Associated with greater complexity, children from these backgrounds may experience worse outcomes.^11 12^ Children with CHD are also more likely to reside in deprived areas.^9^ Deprivation has been linked to poorer CHD outcomes, such as reduced likelihood of completing the Fontan pathway^11^ and infant mortality.^12^ A UK study of postoperative complications found that compared with children of white ethnicity, those of Asian ethnicity had a higher risk of feeding problems and those of black ethnicity had a higher risk of multiple complications.^13^ In the health system of the USA, which differs from the NHS, with care provision variation based on ability to pay, studies found poorer health outcomes with CHD linked to socioeconomic deprivation or minoritised ethnicity or race.^14^,^16^ However, further research is needed to explore this association in a UK context, given that the health system is different.

Given that complications following cardiac surgery are key mediators of long-term outcomes, investigating variation in complication rates by ethnicity and deprivation may help identify underlying inequities in healthcare access, preoperative health status and perioperative care. We hypothesised that the risk of developing important postoperative complications following paediatric cardiac surgery may differ by ethnicity and deprivation and that such variation could contribute to observed differences in mortality following surgery.

## Methods

### Participants and data source

The data source was the National Congenital Heart Disease Audit (NCHDA), which collects information on all children undergoing surgery for CHD across 10 centres in England and Wales. Since 2015, NCHDA has collected information on specific postoperative complications.^17^ Submission of NCHDA data is mandatory and undergoes an annual external validation.

We included all children aged <18 years who underwent cardiac surgery between April 2015 and March 2022 as recorded in the NCHDA database. We created 22 820 ‘30-day episodes’ that started with a patient’s cardiac surgery and ended with the patient’s vital status at 30 days. Surgeries within 30 days for the same patient were counted as part of the initial episode; cardiac surgeries>30 days after the initial surgery were treated as new episodes.

#### Outcome measures

The outcome was the occurrence of a complication within a 30-day postoperative episode. The six defined complications were acute neurological event, unplanned reintervention (unplanned additional cardiac surgery, interventional catheterisation, permanent pacemaker placement or diaphragm plication procedure), renal replacement therapy, extracorporeal life support, necrotising enterocolitis (restricted to children<1 year) and prolonged pleural effusion >10 days. These complications were developed by a multidisciplinary group of healthcare professionals with input from parents and patients.^1^ The detailed definitions (online supplemental table 1) are in the NCHDA data manual.^18^

#### Key exposure variables

Key exposure variables were ethnicity and deprivation as recorded in source data. Ethnicity was categorised as Asian (Bangladeshi, Indian, Pakistani or other), black ethnicity (Caribbean, African or other), multiple, other (Arab, Chinese or other) or white (British, Irish or other). The ethnicity data in NCHDA are taken from the local medical records. The NHS recording of ethnic background in the medical record emphasises self-reporting, which in most cases will mean for children, the reporting is done by their parents.

Deprivation was quantified using the index of multiple deprivation^19^ divided into quintiles with 1 being the least deprived and 5 being the most deprived (Census lower super output) areas. Information on deprivation was only available for children living in England.

#### Preoperative case-mix complexity

We used the following previously described and defined preoperative variables to account for case-mix complexity: sex, age (years) and weight (kg).^20^ Additional preoperative variables (online supplemental table 2) were presence of functionally univentricular heart, level of urgency (elective vs urgent, emergency or salvage) and seven previously described clinical risk variables^21^: presence of acquired comorbidity, additional cardiac risk factors, congenital non-cardiac comorbidity (excluding Down syndrome), congenital cardiac risk factors, Down syndrome, prematurity (<37 weeks gestational age) and severity of illness. 10 specific procedure and 8 diagnostic groups were created according to the prevalence of the relevant complication (online supplemental tables 3 and 4).

### Descriptive statistics

We described the preoperative case mix of our study cohort by the two exposure variables (ethnicity and deprivation) and reported key differences based on χ^2^ tests for categorical variables and the Wilcoxon rank-sum test for continuous variables. We reported the prevalence of each complication outcome based on the exposure variables and we reported key differences. The occurrence of multiple (≧2) complications was cross-tabulated with ethnic group and quintile of deprivation.

### Descriptive models

In prior work, descriptive models that considered preoperative case-mix variables were developed for each of the six complication outcomes for future use in quality assurance. Methods have been previously described^20^ but in brief, the descriptive models were developed by examining the associations between the above 16 candidate preoperative case-mix variables and each of the six complication outcomes separately, using univariable logistic regression, with standard errors clustered by centre and with a significance threshold of p<0.2. Variables meeting this threshold were then entered into a logistic regression model with predictors chosen using backward elimination for each complication outcome, also clustering standard errors by centre. Applying the same p<0.2 threshold, we constructed a multivariable model for each specified outcome.^20^ We report the risk factors independently linked to each complication outcome in online supplemental table 5.

There is a short time interval between surgery and onset of complication for extracorporeal life support and renal replacement therapy,^20^ meaning that death is not considered to be a competing event, and for these complication outcomes, all records were included. For acute neurological event, prolonged pleural effusion, necrotising enterocolitis and unplanned reintervention, the possibility of death as a competing event was present and therefore, we removed the records of patients who died without this complication occurring.^20^

To explore links between ethnicity and deprivation with our study outcomes, these two exposures were separately added into each of the risk prediction models. The results of multivariable logistic regression descriptive models (standard errors clustered by centre) are reported for each of the six complications.

### Missing data

We reported missing variables by complication outcome in all tables and performed complete case analysis in the adjusted models. To conduct a sensitivity analysis for records with missing ethnicity, we assumed values were missing at random and imputed ethnicity using multiple imputation by chained equations, with the imputation model including all variables from the analysis models.

## Results

### Participants and case mix

There were a total of 23 423 30-day postoperative episodes with 1.5% (361) 30-day mortality. Children were a median of 6 months old (IQR 3 months to 4 years) and 55.6% (10 405) were boys. 15.2% (3,559) of children had an acquired comorbidity, 7.5% (1747) had additional cardiac risk factors, 20.8% (4875) had a congenital comorbidity excluding Down syndrome, 1.6% (370) had congenital cardiac risk factors, 7.9% (1842) had Down syndrome, 13.4% (3144) were premature and 14% (3281) had a severity of illness indicator and 32% (7430) of operations were done as an urgent, emergency or salvage procedure.

### Key exposures

We present the demographic and case-mix variables by ethnic background in online supplemental table 6a and based on deprivation quintile for home residence in online supplemental table 6b.

12.7% (2971/23 423) of children in the cohort lived in the least deprived quintile of areas and 26.2% (6143/23 423) resided in the most deprived areas. 11.4% (2678/23 423) of children were of Asian ethnicity, 4.3% (997/23 423) were of Black ethnicity and 62.1% (14 537/23 423) were of white ethnicity. Children of Asian and Black ethnicity were more likely to reside in highly deprived areas (41.6% (1115/2678) and 43.6% (435/997) compared with 21.9% (3183/14 537) of white children living in highly deprived areas.

### Exposures and case mix

**Figure 1:**
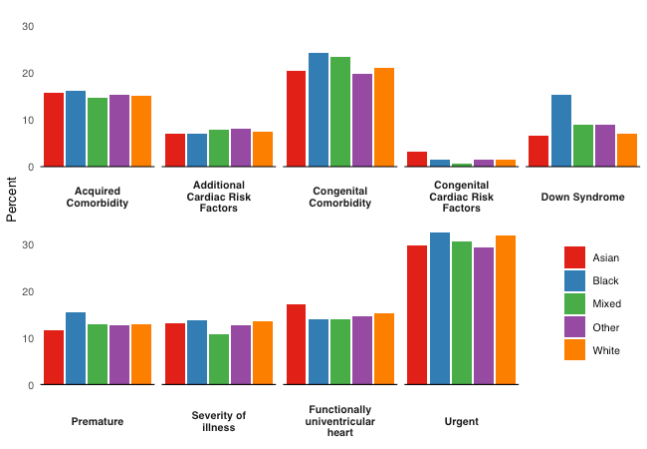
Prevalence of preoperative risk factors by ethnicity

**Figure 2:**
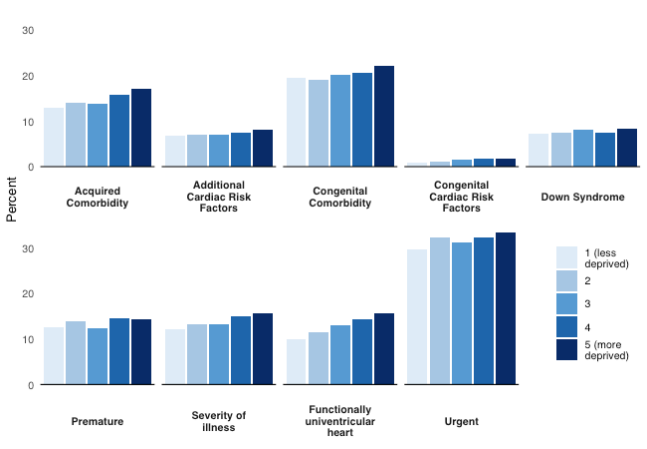
Prevalence of preoperative risk factors by quintile of deprivation.

We depict preoperative case-mix variables by ethnic background in figure 1 and by quintiles of deprivation in figure 2. Children of Asian ethnicity were more likely to have a functionally univentricular heart 17.0% (455/2678) or congenital cardiac risk factors 3.2% (85/2678) than children from the remaining ethnic backgrounds. Children of black ethnicity had a higher prevalence of Down syndrome 15.3% (153/997) and prematurity 15.3% (153/997), compared with children from the remaining ethnic backgrounds.

Children living in the most deprived areas compared with the least deprived areas had the highest prevalence of congenital comorbidity: 22.1% (1359/6143) compared with 19.4% (577/2971); the highest prevalence of a severity of illness indicator 15.6% (961/6143) compared with 12.0% (358/2971); the highest prevalence of functionally univentricular heart 15.6% (960/6143) compared with 9.9% (293/2971); and the highest proportion needing urgent operations 33.4% (2050/6143) compared with 29.5% (876/2971).

### Outcomes

As we report in table 1, procedures were complicated by acute neurological event in 2.2% (526/23 423), unplanned reintervention in 4.3% (1006/23 423), renal replacement therapy in 3.2% (740/23 423), extracorporeal life support in 1.9% (446/23 423), necrotising enterocolitis in 1.9% (257/13 556) and prolonged pleural effusion in 1.3% (158/12 408) of cases. 10.6% (2483/23 423) of children experienced one or more of these complications and 2.2% (524/23 423) of children experienced two or more. Among the cohort of 23 423, two or more complications occurred with higher rates in Asian (70 (2.6%)) and black (27 (2.7%)) children compared with children of multiple ethnicity (10 (1.9%)), other ethnicities (13 (1.3%)) and white ethnicity (347 (2.4%)). Of 2971 children living in the least deprived areas, 193 (6.5%) had one complication and 53 (1.8%) had two or more complications increasing to 518 (8.4%) with one complication and 131 (2.1%) with two or more complications, amongst the 6143 children living in the most deprived areas (table 2).

**Table 1:** Prevalence of defined individual complications and multiple complications by ethnic group

		Total	Asian	Black	Multiple	Other	White	Unknown	P value
		n=23 423	n=2678 (11.4%)	n=997 (4.3%)	n=531 (2.3%)	n=977 (4.2%)	n=14 537 (62.1%)	n=3703 (15.8%)	
Dead at 30 days		361 (1.5%)	50 (1.9%)	22 (2.2%)	5 (0.9%)	12 (1.2%)	204 (1.4%)	68 (1.8%)	0.059
Multiple complications	No complication	20 940 (89.4%)	2362 (88.2%)	898 (90.1%)	471 (88.7%)	880 (90.1%)	12 938 (89.0%)	3391 (91.6%)	<0.001
	1 complication	1959 (8.4%)	246 (9.2%)	72 (7.2%)	50 (9.4%)	84 (8.6%)	1252 (8.6%)	255 (6.9%)	
	≥2 complications	524 (2.2%)	70 (2.6%)	27 (2.7%)	10 (1.9%)	13 (1.3%)	347 (2.4%)	57 (1.5%)	
Acute neurologic event	No	22 897 (97.8%)	2600 (97.1%)	974 (97.7%)	523 (98.5%)	952 (97.4%)	14 201 (97.7%)	3647 (98.5%)	0.005
	Yes	526 (2.2%)	78 (2.9%)	23 (2.3%)	8 (1.5%)	25 (2.6%)	336 (2.3%)	56 (1.5%)	
Unplanned reintervention	No	22 417 (95.7%)	2565 (95.8%)	955 (95.8%)	506 (95.3%)	943 (96.5%)	13 855 (95.3%)	3593 (97.0%)	<0.001
	Yes	1006 (4.3%)	113 (4.2%)	42 (4.2%)	25 (4.7%)	34 (3.5%)	682 (4.7%)	110 (3.0%)	
Renal replacement therapy	No	22 683 (96.8%)	2597 (97.0%)	964 (96.7%)	510 (96.0%)	957 (98.0%)	14 051 (96.7%)	3604 (97.3%)	0.083
	Yes	740 (3.2%)	81 (3.0%)	33 (3.3%)	21 (4.0%)	20 (2.0%)	486 (3.3%)	99 (2.7%)	
Extracorporeal life support	No	22 977 (98.1%)	2625 (98.0%)	976 (97.9%)	527 (99.2%)	964 (98.7%)	14 246 (98.0%)	3639 (98.3%)	0.21
	Yes	446 (1.9%)	53 (2.0%)	21 (2.1%)	4 (0.8%)	13 (1.3%)	291 (2.0%)	64 (1.7%)	
Necrotising enterocolitis	No	13 299 (98.1%)	1440 (98.0%)	555 (98.2%)	318 (97.8%)	543 (96.6%)	8101 (98.3%)	2342 (98.0%)	0.15
	Yes	257 (1.9%)	30 (2.0%)	10 (1.8%)	7 (2.2%)	19 (3.4%)	144 (1.7%)	47 (2.0%)	
Pleural effusion	No	12 250 (98.7%)	1408 (97.0%)	477 (99.0%)	396 (98.0%)	471 (99.6%)	7266 (98.7%)	2232 (100.0%)	<0.001
	Yes	158 (1.3%)	43 (3.0%)	5 (1.0%)	8 (2.0%)	2 (0.4%)	99 (1.3%)	1 (0.0%)	

**Table 2:** Prevalence of defined individual complications and multiple complications by quintile of deprivation (index of multiple deprivation)

		1 (least deprived)	2	3	4	5 (most deprived)	Missing	P value
		n=2971 (12.7%)	n=3240 (13.8%)	n=3867 (16.5%)	n=4601 (19.6%)	n=6143 (26.2%)	n=2601 (11.1%)	
Dead at 30 days		31 (1.0%)	41 (1.3%)	48 (1.2%)	90 (2.0%)	111 (1.8%)	40 (1.5%)	0.005
Multiple complications	No complication	2725 (91.7%)	2982 (89.6%)	3455 (89.4%)	4104 (89.2%)	5494 (89.4%)	2260 (86.9%)	<0.001
	1 complication	193 (6.5%)	273 (8.4%)	331 (8.6%)	390 (8.5%)	518 (8.4%)	254 (9.8%)	
	≥2 complications	53 (1.8%)	65 (2.0%)	81 (2.1%)	107 (2.3%)	131 (2.1%)	87 (3.3%)	
Acute neurologic event	No	2913 (98.0%)	3170 (97.8%)	3785 (97.9%)	4488 (97.5%)	6021 (98.0%)	2520 (96.9%)	0.021
	Yes	58 (2.0%)	70 (2.2%)	82 (2.1%)	113 (2.5%)	122 (2.0%)	81 (3.1%)	
Unplanned reintervention	No	2875 (96.8%)	3107 (95.9%)	3688 (95.4%)	4409 (95.8%)	5885 (95.8%)	2453 (94.3%)	<0.001
	Yes	96 (3.2%)	133 (4.1%)	179 (4.6%)	192 (4.2%)	258 (4.2%)	148 (5.7%)	
Renal replacement therapy	No	2894 (97.4%)	3140 (96.9%)	3748 (96.9%)	4456 (96.8%)	5948 (96.8%)	2497 (96.0%)	0.099
	Yes	77 (2.6%)	100 (3.1%)	119 (3.1%)	145 (3.2%)	195 (3.2%)	104 (4.0%)	
Extracorporeal life support	No	2932 (98.7%)	3176 (98.0%)	3800 (98.3%)	4495 (97.7%)	6031 (98.2%)	2543 (97.8%)	0.036
	Yes	39 (1.3%)	64 (2.0%)	67 (1.7%)	106 (2.3%)	112 (1.8%)	58 (2.2%)	
Necrotising enterocolitis	No	1618 (98.2%)	1842 (98.2%)	2215 (98.3%)	2745 (98%)	3489 (98.2%)	1390 (97.6%)	0.752
	Yes	29 (1.8%)	34 (1.8%)	39 (1.7%)	57 (2.0%)	69 (1.8%)	41 (2.4%)	
Pleural effusion	No	1576 (99.5%)	1719 (99.1%)	2032 (98.7%)	2468 (99.1%)	3207 (98.3%)	1248 (97.8%)	<0.001
	Yes	8 (0.5%)	15 (0.9%)	27 (1.3%)	23 (0.9%)	57 (1.7%)	28 (2.2%)	

### Case-mix adjusted outcomes

We present the univariable and multivariable associations of ethnicity and deprivation categories with each complication outcome in table 3.

**Table 3:** Univariable and adjusted association of ethnicity and deprivation with the occurrence of complications

		Acute neurological event				Unplanned reintervention				Renal replacement therapy			
		OR (95% CI)	P value	aOR (95% CI)	P value	OR (95% CI)	P value	aOR (95% CI)	P value	OR (95% CI)	P value	aOR (95% CI)	P value
Ethnicity	White	Ref		Ref	0.379	Ref		Ref	0.309	Ref		Ref	**0.022**
	Asian	1.27 (0.88 to 1.84)	0.197	1.23 (0.86 to 1.76)		0.90 (0.66 to 1.23)	0.51	0.87 (0.63 to 1.19)		0.90 (0.69 to 1.17)	0.437	0.90 (0.68 to 1.20)	
	Black	1.00 (0.57 to 1.77)	0.99	1.06 (0.59 to 1.92)		0.90 (0.60 to 1.35)	0.604	0.91 (0.62 to 1.34)		0.99 (0.62 to 1.59)	0.966	1.07 (0.67 to 1.70)	
	Multiple	0.64 (0.37 to 1.13)	0.126	0.67 (0.40 to 1.12)		1.00 (0.66 to 1.53)	1	1.05 (0.67 to 1.64)		1.19 (0.76 to 1.85)	0.44	1.26 (0.83 to 1.94)	
	Other	1.11 (0.60 to 2.05)	0.742	1.14 (0.57 to 2.25)		0.73 (0.55 to 0.98)	**0.033**	0.75 (0.57 to 1.00)		0.60 (0.40 to 0.92)	**0.02**	0.59 (0.40 to 0.87)	
IMD quintile	1 (least deprived)	Ref		Ref	**0.05**	Ref		Ref	**0.037**	Ref		Ref	0.806
	2	1.11 (0.73 to 1.70)	0.623	1.05 (0.69 to 1.60)		1.29 (1.02 to 1.62)	**0.036**	1.25 (0.99 to 1.57)		1.20 (0.88 to 1.62)	0.245	1.17 (0.85 to 1.61)	
	3	1.09 (0.86 to 1.39)	0.472	1.01 (0.77 to 1.31)		1.46 (1.17 to 1.82)	**<0.001**	1.38 (1.11 to 1.70)		1.19 (1.01 to 1.41)	**0.041**	1.11 (0.88 to 1.39)	
	4	1.27 (0.85 to 1.90)	0.235	1.14 (0.76 to 1.70)		1.31 (1.00 to 1.72)	**0.047**	1.18 (0.88 to 1.59)		1.22 (0.96 to 1.56)	0.108	1.10 (0.82 to 1.47)	
	5 (most deprived)	1.03 (0.79 to 1.33)	0.845	0.83 (0.64 to 1.07)		1.32 (0.97 to 1.81)	**0.077**	1.12 (0.82 to 1.54)		1.23 (1.03 to 1.47)	**0.022**	1.03 (0.80 to 1.33)	
		**Extracorporeal life support**				**Necrotising enterocolitis**				**Prolonged pleural effusion**			
		**OR (95% CI)**	**P value**	**aOR (95% CI)**	**P value**	**OR (95% CI)**	**P value**	**aOR (95% CI)**	**P value**	**OR (95% CI)**	**P value**	**aOR (95% CI)**	**P value**
Ethnicity	White	Ref		Ref	**0.001**	Ref		Ref	**0.004**	Ref		Ref	**0.027**
	Asian	0.99 (0.73 to 1.35)	0.941	0.98 (0.69 to 1.41)		1.18 (0.86 to 1.62)	0.301	1.03 (0.71 to 1.49)		2.25 (1.11 to 4.56)	**0.025**	1.74 (0.93 to 3.25)	
	Black	1.05 (0.65 to 1.72)	0.835	1.20 (0.80 to 1.79)		1.03 (0.39 to 2.69)	0.956	1.12 (0.43 to 2.94)		0.77 (0.41 to 1.43)	0.407	0.69 (0.36 to 1.33)	
	Multiple	0.37 (0.19 to 0.72)	**0.003**	0.41 (0.21 to 0.82)		1.23 (0.43 to 3.50)	0.699	1.26 (0.45 to 3.52)		1.47 (0.91 to 2.36)	0.114	1.32 (0.82 to 2.12)	
	Other	0.66 (0.41 to 1.07)	0.091	0.70 (0.43 to 1.15)		1.97 (1.38 to 2.80)	**<0.001**	1.83 (1.22 to 2.75)		0.31 (0.10 to 0.94)	**0.038**	0.32 (0.10 to 1.06)	
IMD quintile	1 (least deprived)	Ref		Ref	**0.001**	Ref		Ref	0.609	Ref		Ref	**0.046**
	2	1.51 (1.07 to 2.14)	**0.019**	1.45 (0.99 to 2.12)		1.04 (0.67 to 1.61)	0.875	1.04 (0.66 to 1.63)		1.72 (0.74 to 3.97)	0.204	1.66 (0.65 to 4.25)	
	3	1.33 (1.00 to 1.76)	0.053	1.25 (0.89 to 1.77)		0.99 (0.67 to 1.46)	0.952	0.91 (0.58 to 1.44)		2.61 (1.10 to 6.22)	**0.03**	2.30 (0.85 to 6.24)	
	4	1.77 (1.45 to 2.16)	**<0.001**	1.56 (1.12 to 2.16)		1.15 (0.92 to 1.45)	0.221	1.07 (0.84 to 1.35)		1.85 (0.55 to 6.19)	0.32	1.69 (0.50 to 5.77)	
	5 (most deprived)	1.40 (1.05 to 1.86)	**0.023**	1.12 (0.75 to 1.65)		1.05 (0.86 to 1.29)	0.616	0.91 (0.73 to 1.14)		3.52 (1.51 to 8.20)	**0.003**	2.84 (1.09 to 7.40)	

aOR, adjusted odds ratio; OR, odds ratio.

#### Acute neurological event

There was no evidence of association between ethnicity and acute neurological event. Children in deprivation quintiles 1 and 5 had slightly lower adjusted OR (aOR) of acute neurological event than children in quintiles 2–4 (p=0.05).

#### Unplanned reintervention

There was no evidence of association between ethnicity and unplanned reintervention. Children living in more deprived areas had increased odds of unplanned reintervention compared with children living in the less deprived areas in the univariable analysis. In the adjusted model, the aORs for quintiles 2–5 were slightly higher than aOR for quintile 1 (least deprived) p value=0.04.

#### Renal replacement therapy

There was some evidence of reduced adjusted odds of renal replacement therapy in children of other compared with white ethnicity aOR 0.59 (95% CI 0.40 to 0.87; p=0.02). Although there was some evidence of increased odds of renal replacement therapy with increasing levels of deprivation in the univariable analysis, there was no evidence of association in the adjusted analysis.

#### Extracorporeal life support

There was evidence of reduced adjusted odds of extracorporeal life support in children of multiple compared with white ethnicity aOR 0.41 (95% CI 0.21 to 0.82 p=0.001). There was strong evidence for higher adjusted odds of extracorporeal life support with deprivation: quintiles 2–5 were significantly higher than aOR for quintile 1 (least deprived) p value=0.001.

#### Necrotising enterocolitis

There was some evidence of increased adjusted odds of necrotising enterocolitis in children of other compared with white ethnicity, aOR 1.83 (95% CI 1.22 to 2.75 p=0.004). There was no evidence of association between deprivation and necrotising enterocolitis.

#### Prolonged pleural effusion

There was some evidence of increased adjusted odds of prolonged pleural effusion with Asian compared with white ethnicity aOR 1.74 (95% CI 0.93 to 3.25) p=0.03. There was evidence of increasing adjusted odds of prolonged pleural effusion with deprivation: aOR for high versus low deprivation 2.84 (95% CI 1.09 to 7.40).

#### Missing data

15.8% (3703/23 423) of episodes were missing data on ethnicity and 11.1% (2601/23 423) were missing data on deprivation; these records were not included in the models (complete case analysis). When restricting the cohort to children living in England, data were missing on deprivation in 1.6% (336/21 158) cases. The sensitivity analysis was consistent with the primary analysis (online supplemental table 7).

## Discussion

### Key findings

The study cohort included all children who underwent cardiac surgery in England and Wales over a 7-year period. Only 12.7% lived in the least deprived areas, and 62.1% were of white ethnic background—both proportions were lower than expected when compared with the general child population.^22 23^ Nearly half of the children from minoritised ethnic backgrounds resided in the most deprived areas. Most characteristics of high case-mix complexity rose in prevalence with increasing level of area deprivation; and selected case-mix factors (eg, functionally univentricular heart and Asian ethnicity) were more common in children with minoritised ethnic backgrounds. Since case complexity is linked to the complication outcomes, we looked at the adjusted analyses from our modelling: these suggested a link between increasing levels of area deprivation and the complication outcomes of unplanned reintervention, extracorporeal life support and prolonged pleural effusion. The relationships between ethnic background and the occurrence of complications were less clear.

### Research in context

Studies based in the USA have indicated that lower socioeconomic status, black and Hispanic ethnic origin are risk factors for mortality and unplanned hospital re-admission following CHD surgery.^14 24^ These disparities have been attributed to structural inequities in the healthcare system and financial barriers faced by families.^25^ A study of children undergoing CHD surgery in China found that low and middle family socioeconomic status was associated with poorer outcomes than high family socioeconomic status.^26^ A study based in India reported severe financial stress for families linked to a child undergoing CHD surgery.^27^ Healthcare in the UK National Health Service is universally available and free at the point of access; nonetheless, in England, the key childhood indicators of neonatal and infant mortality showed widening inequalities in 2023–2024.^28^ These concerning trends are mirrored by the national audit data related to infants with CHD: those treated between 2005 and 2009 had no outcome differences based on quintiles of deprivation,^29^ whereas among those born between 2018 and 2022 those resident in high deprivation areas had increased infant mortality.^12^ Since families living in the most deprived areas in England, and families of black and Asian ethnicities are more likely to have a child affected by CHD than families residing in low deprivation areas and of white ethnicity^9^; as has been reported in other regions,^30^,^32^ it is particularly important to explore the links between these social factors and poorer outcomes to elucidate pathways to improve. Our study focused specifically on perioperative care and the key metric of postoperative complications.^3 33^ In unadjusted analyses, multiple complications, a very adverse postoperative outcome,^6^ were more likely based on Asian or black ethnic background and higher levels of area deprivation. Our interpretation of this is limited since case-mix adjustment was only available for specific individual complications and noting that children of Asian and black backgrounds are more likely to live in areas of high deprivation. Children of multiple ethnicity had lower odds of extracorporeal membrane oxygenation, and children of Asian ethnicity had higher odds of prolonged pleural effusion; one possible explanation is case-mix differences that are not accounted for in our study.

Prior research has shown that postoperative extracorporeal life support is more likely to be needed in children who were sicker before surgery,^34^ around half of children who need extracorporeal life support after cardiac surgery die before discharge^34^ and survivors may experience long-term impacts.^7 35^ Our analysis suggested this complication was more likely with higher levels of area deprivation, and one explanation for this is these children had poorer health going into their surgery. Poorer health before surgery is also a potential explanation for the occurrence of a more challenging operation, leading to worse technical results,^36 37^ thus contributing to a higher risk of reintervention being needed.

Postoperative prolonged pleural effusion is more likely in the winter,^38^ which has been explained by higher rates of respiratory infections. Significant paediatric respiratory infections are more likely to occur with poorer living conditions, for example, over-crowded housing, which might explain the link that we observed between higher area deprivation levels and prolonged pleural effusion after children’s heart surgery.^39^

### Strengths and weaknesses

A strength of our study is that it used a large population-based dataset consisting of validated national audit data. Weaknesses of our study related to data quality: first, the complication outcomes were added as new mandatory data items in 2015. Their definitions were added to the NCHDA manual, based on the prospective pilot study which ran in five centres, 2015–2017, and the complications were externally validated alongside other data items in the audit.^1^,^3^ Nonetheless, certain complications are complex measures with potential for data quality issues as centres learn the new measures. Although the standard errors for the multivariable descriptive models were adjusted for clustering by centre, this is unlikely to fully account for all intercentre differences, which are complex and may involve practice preferences, team performance, intercentre variation in case complexity and the social factors within the local population. There was a large amount of missing data on ethnicity (15.6%) and deprivation (11%), and we were unable to consider the ethnic groups disaggregated into subcategories, given the way these were captured in the source data. For example, we were unable to consider subgroups of Asian ethnicity, noting that a higher proportion of children of Asian Pakistani and Asian Bangladeshi ethnicity were living in the most deprived areas versus Asian Indian ethnicity.^40^

Then our study is further limited by the scope of the data items with the risk variables restricted to those collected for the national audit.^20^

### Implications for research and care

Further research is needed to explore the relationship between deprivation, ethnic backgrounds and perioperative outcomes in CHD. Given that poorer preoperative condition is linked to a more challenging postoperative course, the optimisation of a child’s condition in a hospital setting may be required if this cannot be undertaken at home, based on poor housing related to socioeconomic deprivation.

## Conclusions

Greater area deprivation in England is associated with increased preoperative medical complexity and a higher incidence of certain postoperative complications among children with CHD. Further research is needed to explore the relationship between ethnic background and perioperative outcomes in CHD and to develop pathways to improvement based on social factors.

## Supplementary material

10.1136/archdischild-2025-329267 online supplemental file 1

## Data Availability

Data may be obtained from a third party and are not publicly available.
