# Supplementary material for: Postoperative complications after paediatric cardiac surgery: the role of ethnicity and deprivation – a national cohort study
Source: Arch Dis Child. 2025 Dec 13;111(4):e329267. doi: 10.1136/archdischild-2025-329267 (PMC13018760; doi:10.1136/archdischild-2025-329267)
Supplement: online supplemental file 1 [file archdischild-111-4-s001.docx]

# Supplementary Table 1: Definitions of Complication Outcomes

[Supporting dataset documentation - NICOR](https://www.nicor.org.uk/datasets/supporting-data-set-documentation)

## Acute Neurological Event

Complication codes ‘European Paediatric Cardiac Code (EPCC) code^[[1]](#footnote-1)^ 158399 - Acute neurological event within 30 days after cardiovascular procedure’, ‘EPCC code 158257 - New neurological impairment (global or focal) present at discharge’ or ‘Post procedure seizures’

| **Acute neurological event (ACUTE NEUROLOGICAL EVENT)**  We recommend that children who have undergone a cardiovascular procedure have a clinical assessment as they recover in the first 48 hours and longer for those with slower recovery given that the timeline for ascertainment is up to 30 days after the procedure. If there are any clinical concerns from the bedside as to the possibility of an acute neurological event please use the definition below. | |
| --- | --- |
| **Timeline details** | **Definition criteria** |
| • Includes neurological morbidities that, based on best clinical judgement, arose as new findings around the time of cardiovascular procedure that were detected within 30 days of the procedure.  • It is recognised that in certain circumstances such as where a child is very sick on life support, pre-procedure assessment is challenging, in these circumstances as full an evaluation as possible to be completed, incorporating serial assessments over time.  • Children may have neurological events prior to cardiovascular procedure and if this is the case please use (102012. Pre-procedural neurological impairment, 102013. Preprocedural cerebral abnormality on imaging, 102018. Preprocedural seizures) these are not to be included. | **Any or all of the following arising de novo post-procedure:**  A) Physical signs of neurological injury as diagnosed by a neurologist: focal neurological deficit (includes cranial nerve deficits, hemiplegia and monoplegia), brain death, prolonged coma or significantly altered conscious level after cessation of sedatives, spinal cord ischaemia leading to impaired function, basal ganglia damage or brain stem injury leading to abnormal cough or gag reflex.  B) Brain imaging reported by a neuroradiologist: a new abnormality on either cranial ultrasound, CT scan or MRI scan including: intracranial haemorrhage, extra axial haemorrhage, stroke, white matter damage, hypoxic ischaemic brain injury or uncal herniation.  C) Abnormal movements as diagnosed by a neurologist: seizures requiring medical therapy to control them or new persistent movement disorder including choreiform or athetoid movement. |

## Prolonged Pleural Effusion or Chylothorax

Complication code ‘EPCC code 158065 - Postprocedural prolonged pleural drainage (over 10 days)’

| **Prolonged pleural effusion or chylothorax** | |
| --- | --- |
| **Timeline details**  Prolonged pleural effusion is a postprocedural effusion or a chylothorax with duration greater than 10 days. The diagnosis of prolonged pleural effusion or chylothorax is made from after surgery and within 30 days after the procedure. | **Definition criteria**  The emphasis is on the duration of drainage. Detecting chyle in pleural, peritoneal or pericardial fluid does not count as a complication unless it is associated with prolonged drainage >10 continuous days.  This includes chylous pleural effusion or significant chylous pericardial effusion or significant chylous ascites or a prolonged non-chylous effusion that requires thoracic drainage at least 10 days following index cardiac surgery.  Chylous effusions are characterised by milky appearance and a pleural fluid white blood cell count of >1000 cells/μl with lymphocytes >80%. If the child is on normal feeds the triglyceride level in the pleural fluid will be >1.1 mmol/L or the ratio between the pleural triglyceride level and the serum triglyceride level will exceed 1 |

## Extracorporeal Life Support

Complication code ‘EPCC code 150009 - Requirement for mechanical circulatory support

| **Extracorporeal life support** | |
| --- | --- |
| **Timeline details**  Extracorporeal life support within 30 days following a procedure, including the rare cases when a child was on extracorporeal life support before surgery.  Preoperative Extracorporeal life support is a risk factor for surgery captured by the risk stratification score.  When we report this complication for the audit, the patients who had preoperative Extracorporeal life support can be considered in the analysis as this is a known risk factor for post operative Extracorporeal life support. | **Definition criteria**  This morbidity is defined by the presence of an extracorporeal life support system connected to the patient following the operation, whether it was placed in the operating theatre or in the intensive care unit (ICU), and whether the indication was cardiac arrest, low cardiac output state, poor cardiac function, arrhythmia, residual or recurrent cardiac lesion, pulmonary including pulmonary hypertension or sepsis.  Only post-cardiotomy Extracorporeal life support is included in this complication.  Additional complications arising on Extracorporeal life support need to be captured in addition within the audit data. This applies for example to acute kidney injury (AKI) (defined as renal support on Extracorporeal life support – for renal failure and fluid management), acute neurological event, unplanned reinterventions. It should not apply to bleeding /chest reopening (defined as ongoing care). |

## Necrotising Enterocolitis

Complication code ‘EPCC code 158375- Postprocedural necrotising enterocolitis - established requiring treatment’.

| **Necrotising enterocolitis** | |
| --- | --- |
| **Timeline details** | **Definition criteria** |
| Necrotising enterocolitis as a new diagnosis from after surgery until 30 days following the procedure.  Should not include patients with preoperative diagnosis of Necrotising enterocolitis. Patients who have preoperative Necrotising enterocolitis will be recorded in NCHDA dataset as a preoperative condition (this is one of the preoperative comorbidities in the NCHDA dataset) and as such are not postoperative cases. Post-operative Necrotising enterocolitis in a child who had pre-operative Necrotising enterocolitis within 30 days is the continuation of the same condition.  There is no requirement for grading. As a practical guide, however, the following simplified classification has been suggested. Moderate - any child meeting the criteria who does not need surgery and survives; severe - a child with Necrotising enterocolitis who needs surgery and/or dies. | **Systemic signs** include temperature instability, apnoea, bradycardia, raised inflammatory markers, thrombocytopenia, shock features. In Necrotising enterocolitis these are present with abdominal and or radiological signs stated below.  **Abdominal - Intestinal signs** include abdominal distension, reduced or absent bowel sounds, larger than normal gastric aspirates, gastric bleeding, rectal bleeding, abdominal tenderness or cellulitis.  A child who develops only mild systemic and or abdominal-intestinal signs and is treated with only with a 24-48 hour rule out course of nil by mouth (NBM) and antibiotics followed by re-starting feeds based on improvement should not be counted.  If a general surgeon assesses the child and based on features from the systemic signs and abdominal signs elects to treat the child as Necrotising enterocolitis for minimum 5days then this case should be counted.  Any child with a surgical abdomen who has a more serious picture – perforation, peritonitis, abdominal mass, is to be counted.  **Radiological signs**  The following are radiological signs of Necrotising enterocolitis and cases with these should be counted: pneumatosis coli, portal gas, perforation – pneumoperitoneum (excluding air under the diaphragm associated with insertion of a peritoneal catheter in theatre or in ICU, or accidental opening of the peritoneum in the operation). |

## Unplanned Reintervention

Type 1 Complication code ‘EPCC code 124307- Unplanned reoperation/ reintervention within 30 days of procedure (excludes bleeding)’. Type 2 ‘EPCC code 110633- Postprocedural complete atrioventricular block requiring permanent pacemaker system’ and Type 3 ‘EPCC code 158190- Phrenic nerve injury requiring plication of diaphragm’.

| **Unplanned reoperation or reintervention** | |
| --- | --- |
| **Timeline details**  Unplanned re-interventions are procedures outside the expected patient pathway, which may be undertaken at any time from the start of the postoperative admission up until 30 days following the procedure. Additional procedures or revisions undertaken within the primary trip to the operating theatre (incorporating return onto cardiopulmonary bypass) are not included in the definition of re-operation.  Procedures (catheters or operations) that were planned prior to the surgery being undertaken are not to be included. | **Definition criteria**  Type 1  Unplanned return to the operating room or cardiac catheter laboratory for a cardiac intervention within 30 days for procedures that were not intended in the planning phase, follow an initial primary cardiac surgery and result in “substantive alteration to heart” - incorporating cardiac bypass, cardiac non-bypass, interventional catheterisation.  **Excludes:** diagnostic catheters; interventional catheters that were planned preoperatively; delayed chest closure; procedures for bleeding; mechanical circulatory support procedures; wound procedures carried out in theatre (captured separately); non-cardiac surgery procedures.  Type 2  Unplanned permanent pacemaker placement as separate category  Type 3  Diaphragm plication as separate category |

## Need for Renal Replacement Therapy

Complication code ‘EPCC code 158213- Acute kidney injury requiring dialysis’.

| **Need for renal replacement therapy for renal impairment / failure and or systemic inflammatory response** | |
| --- | --- |
| **Timeline details**  Includes renal replacement therapy when initiated as a new support at any time from the start of the postoperative admission to ICU up until 30 days following the procedure. | **Definition criteria**  The child requires renal replacement therapy  With modalities of either peritoneal dialysis or haemo-filtration for renal failure (oligo-anuria of <0.5 ml/kg/hour and elevated creatinine level for age) and or fluid  overload which may be related to systemic inflammatory response. |

# Supplementary Table 2: Definitions of additional risk factors

[Supporting dataset documentation - NICOR](https://www.nicor.org.uk/datasets/supporting-data-set-documentation)

| **NCHDA CO-MORBIDITY CODES** | **Additional Risk Factor Group** |
| --- | --- |
| 101351. Pulmonary embolism | Acquired Comorbidity |
| 101400. Secondary systemic hypertension | Acquired Comorbidity |
| 101401. Systemic hypertension | Acquired Comorbidity |
| 101402. Primary (essential) systemic hypertension | Acquired Comorbidity |
| 101404. Systemic hypertension due to aortic arch obstruction | Acquired Comorbidity |
| 101501. Persistent pulmonary hypertension of the newborn (PFC) | Acquired Comorbidity |
| 101505. Necrotising enterocolitis | Acquired Comorbidity |
| 101512. Meconium aspiration | Acquired Comorbidity |
| 102006. Preprocedural coagulation disorder | Acquired Comorbidity |
| 102007. Preprocedural renal failure (creatinine over 1.5 times uProlonged pleural effusionr limit of normal for age) | Acquired Comorbidity |
| 102008. Preprocedural renal failure requiring dialysis | Acquired Comorbidity |
| 102009. Preprocedural septicaemia | Acquired Comorbidity |
| 102012. Preprocedural neurological impairment | Acquired Comorbidity |
| 102013. Preprocedural cerebral abnormality on imaging | Acquired Comorbidity |
| 102017. Preprocedural tracheostomy | Acquired Comorbidity |
| 102018. Preprocedural seizures | Acquired Comorbidity |
| 102036. Preprocedural hepatic dysfunction | Acquired Comorbidity |
| 102037. Preprocedural respiratory syncytial virus (RSV) infection | Acquired Comorbidity |
| 102038. Preprocedural necrotising enterocolitis: treated medically | Acquired Comorbidity |
| 102039. Preprocedural necrotising enterocolitis: treated surgically | Acquired Comorbidity |
| 140305. Psychomotor developmental delay | Acquired Comorbidity |
| 140340. Brain abscess | Acquired Comorbidity |
| 140342. Cerebrovascular accident (stroke) | Acquired Comorbidity |
| 140372. Anoxic-ischaemic encephalopathy | Acquired Comorbidity |
| 140375. Hyperthyroidism | Acquired Comorbidity |
| 140390. Diabetes mellitus | Acquired Comorbidity |
| 140438. Sleep related breathing disorder | Acquired Comorbidity |
| 140494. Diabetes mellitus: requiring insulin | Acquired Comorbidity |
| 140495. Diabetes mellitus: on oral therapy | Acquired Comorbidity |
| 140496. Diabetes mellitus: managed with diet alone | Acquired Comorbidity |
| 140565. Meningitis | Acquired Comorbidity |
| 158210. Kidney failure | Acquired Comorbidity |
| 160302. Lower respiratory tract infection | Acquired Comorbidity |
| 160305. Lung disease | Acquired Comorbidity |
| 160310. Asthma | Acquired Comorbidity |
| 160800. Acquired bronchial disease | Acquired Comorbidity |
| 160900. Airway disease | Acquired Comorbidity |
| 161320. Diaphragm paralysis | Acquired Comorbidity |
| 030102. Visceral heterotaxy (abnormal arrangement thoraco-abdominal organs) | Congenital Comorbidity |
| 030109. Position or morphology of thoraco-abdominal organs abnormal | Congenital Comorbidity |
| 030209. Lung anomaly | Congenital Comorbidity |
| 030214. Functionally congenital single lung | Congenital Comorbidity |
| 030305. Tracheobronchial anomaly | Congenital Comorbidity |
| 030603. Intestines malrotated | Congenital Comorbidity |
| 102049. Preprocedural gastrostomy present | Congenital Comorbidity |
| 102304. Hereditary disorder potentially with associated heart disease | Congenital Comorbidity |
| 140101. Chromosomal anomaly | Congenital Comorbidity |
| 140103. Trisomy 18: Edwards' syndrome | Congenital Comorbidity |
| 140104. Trisomy 13: Patau's syndrome | Congenital Comorbidity |
| 140105. 45XO: Turner’s syndrome | Congenital Comorbidity |
| 140120. Gene mutation or deletion | Congenital Comorbidity |
| 140121. 22q11 microdeletion | Congenital Comorbidity |
| 140200. Syndrome-association potentially with cardiac involvement | Congenital Comorbidity |
| 140206. 22q11 microdeletion with full DiGeorge sequence (including immune dysfunction) | Congenital Comorbidity |
| 140210. Friedreich’s ataxia | Congenital Comorbidity |
| 140217. Marfan syndrome | Congenital Comorbidity |
| 140219. Noonan syndrome | Congenital Comorbidity |
| 140221. Pompe’s disease: glycogen storage disease type IIa | Congenital Comorbidity |
| 140228. Tuberous sclerosis | Congenital Comorbidity |
| 140230. Williams syndrome (infantile hypercalcaemia) | Congenital Comorbidity |
| 140232. Fetal rubella syndrome | Congenital Comorbidity |
| 140234. Duchenne’s muscular dystrophy | Congenital Comorbidity |
| 140258. Muscular dystrophy | Congenital Comorbidity |
| 140262. Ehlers-Danlos syndrome | Congenital Comorbidity |
| 140266. Alagille syndrome: arteriohepatic dysplasia | Congenital Comorbidity |
| 140300. Noncardiac abnormality potentially with associated heart disease | Congenital Comorbidity |
| 140304. Non-cardiothoracic-vascular abnormality | Congenital Comorbidity |
| 140306. Cystic fibrosis | Congenital Comorbidity |
| 140307. Congenital diaphragmatic hernia | Congenital Comorbidity |
| 140308. Tracheo-oesophageal fistula | Congenital Comorbidity |
| 140310. Omphalocoele (exomphalos) | Congenital Comorbidity |
| 140311. Duodenal stenosis/atresia | Congenital Comorbidity |
| 140321. Sickle cell disease | Congenital Comorbidity |
| 140323. Renal abnormality | Congenital Comorbidity |
| 140328. Congenital coagulation disorder | Congenital Comorbidity |
| 140329. Thoracic-mediastinal abnormality | Congenital Comorbidity |
| 140333. Microcephaly | Congenital Comorbidity |
| 140347. Choanal atresia | Congenital Comorbidity |
| 140349. Tracheobronchial malacia | Congenital Comorbidity |
| 140352. Hypothyroidism | Congenital Comorbidity |
| 140391. Cerebral anomaly | Congenital Comorbidity |
| 140392. Connective tissue disease | Congenital Comorbidity |
| 140409. Kyphoscoliosis | Congenital Comorbidity |
| 140412. Cleft lip or palate | Congenital Comorbidity |
| 140415. Scoliosis | Congenital Comorbidity |
| 140485. Loeys-Dietz Syndrome (transforming growth factor beta receptor (TGFBR) gene mutation) | Congenital Comorbidity |
| 140490. Von Willebrand disease | Congenital Comorbidity |
| 140540. Maternally derived fetal disease or syndrome potentially with associated heart disease | Congenital Comorbidity |
| 140550. Major anomaly of gastrointestinal system | Congenital Comorbidity |
| 140601. Multiple congenital malformations | Congenital Comorbidity |
| 161001. Tracheal stenosis | Congenital Comorbidity |
| 161009. Tracheal disease | Congenital Comorbidity |
| 101723. Shock | Severity of illness |
| 102002. Preprocedural shock | Severity of illness |
| 102005. Preprocedural acidosis | Severity of illness |
| 102014. Preprocedural mechanical ventilatory support | Severity of illness |
| 102015. Preprocedural mechanical circulatory support | Severity of illness |
| 102031. Preprocedural shock at time of surgery (persistent) | Severity of illness |
| 102033. Preprocedural cardiopulmonary resuscitation (less than 48 hours) | Severity of illness |
| 102050. Preprocedural inotropic support therapy | Severity of illness |
| 110021. Cardiac arrest | Severity of illness |
| 163001. Respiratory failure | Severity of illness |
| 102202. Premature birth | Premature |
| 102205. Premature birth 32-35 weeks | Premature |
| 102206. Premature birth less than 32 weeks | Premature |
| 140102. Trisomy 21: Down’s syndrome | Downs Syndrome |
| 070001. Ventricular dyssynchrony | Additional Cardiac Risk Factors |
| 070111. Right ventricular dysfunction | Additional Cardiac Risk Factors |
| 070610. Left ventricular dysfunction | Additional Cardiac Risk Factors |
| 100600. Endocarditis | Additional Cardiac Risk Factors |
| 100601. Infective endocarditis | Additional Cardiac Risk Factors |
| 100608. Infective endocarditis of tricuspid valve | Additional Cardiac Risk Factors |
| 100609. Infective endocarditis of pulmonary valve | Additional Cardiac Risk Factors |
| 100610. Infective endocarditis of mitral valve | Additional Cardiac Risk Factors |
| 100611. Infective endocarditis of aortic valve | Additional Cardiac Risk Factors |
| 100612. Endocarditis of right atrioventricular valve | Additional Cardiac Risk Factors |
| 100613. Endocarditis of left atrioventricular valve | Additional Cardiac Risk Factors |
| 100614. Endocarditis of common atrioventricular valve | Additional Cardiac Risk Factors |
| 100618. Endarteritis at site of arterial duct | Additional Cardiac Risk Factors |
| 100620. Heart abscess | Additional Cardiac Risk Factors |
| 100627. Aortic perivalvar (root) abscess | Additional Cardiac Risk Factors |
| 100641. Bacterial endocarditis | Additional Cardiac Risk Factors |
| 100664. Postprocedural endocarditis | Additional Cardiac Risk Factors |
| 100665. Preprocedural endocarditis | Additional Cardiac Risk Factors |
| 100666. Endarteritis at site of aortic coarctation intervention | Additional Cardiac Risk Factors |
| 100688. Endocarditis of truncal valve or neo-aortic valve of truncal origin | Additional Cardiac Risk Factors |
| 100689. Endocarditis of neopulmonary valve | Additional Cardiac Risk Factors |
| 100690. Endocarditis of the neo-aortic valve of pulmonary origin | Additional Cardiac Risk Factors |
| 100740. Dilated cardiomyopathy due to congenital heart disease | Additional Cardiac Risk Factors |
| 100930. Ischaemic heart disease | Additional Cardiac Risk Factors |
| 100953. Chronic total occlusion of coronary artery | Additional Cardiac Risk Factors |
| 101012. Endocardial fibroelastosis | Additional Cardiac Risk Factors |
| 101301. Pulmonary arterial hypertension | Additional Cardiac Risk Factors |
| 101302. Idiopathic (primary) pulmonary hypertension | Additional Cardiac Risk Factors |
| 101306. Pulmonary vascular disease | Additional Cardiac Risk Factors |
| 101308. Irreversible pulmonary vascular disease due to congenital heart disease (Eisenmenger Syndrome) | Additional Cardiac Risk Factors |
| 101320. Secondary pulmonary hypertension | Additional Cardiac Risk Factors |
| 101321. Pulmonary hypertension due to congenital systemic-to-pulmonary shunt | Additional Cardiac Risk Factors |
| 101363. Elevated lung resistance for biventricular repair (over 6 Wood units) | Additional Cardiac Risk Factors |
| 101364. Elevated lung resistance for heart transplant (over 4 Wood units) | Additional Cardiac Risk Factors |
| 101365. Elevated lung resistance for univentricular repair (over 2 Wood units) | Additional Cardiac Risk Factors |
| 101510. Transient myocardial ischaemia | Additional Cardiac Risk Factors |
| 101800. Myocardial infarction | Additional Cardiac Risk Factors |
| 101801. Acute myocardial infarction | Additional Cardiac Risk Factors |
| 101826. Acute coronary syndrome: myocardial infarct with ST elevation (STEMI) | Additional Cardiac Risk Factors |
| 101827. Acute coronary syndrome: myocardial infarct without ST elevation (NSTEMI) | Additional Cardiac Risk Factors |
| 102016. Preprocedural pulmonary hypertension | Additional Cardiac Risk Factors |
| 102034. Preprocedural myocardial dysfunction | Additional Cardiac Risk Factors |
| 102045. Preprocedural pulmonary hypertension (pulmonary pressure more than or equal to systemic pressure): echo data | Additional Cardiac Risk Factors |
| 102046. Preprocedural pulmonary hypertension (pulmonary pressure more than or equal to systemic pressure): catheter data | Additional Cardiac Risk Factors |
| 152231. Residual pulmonary hypertension after relief of systemic-to-pulmonary shunt | Additional Cardiac Risk Factors |

## Supplementary table 3: Congenital heart disease (CHD) diagnosis as defined and grouped by UK National Congenital Heart Diseases Audit (NCHDA), ranked by prevalence for each complication and the CHD groups used for complication risk prediction models.

[Supporting dataset documentation - NICOR](https://www.nicor.org.uk/datasets/supporting-data-set-documentation)

|  | **CHD Type** | **Prevalence range (%)** | **Complication prevalence group** |
| --- | --- | --- | --- |
| Necrotising enterocolitis | Subaortic stenosis, Aortic regurgitation, Interatrial communication (ASD), Vascular ring, Miscellaneous congenital heart disease (CHD) terms, Arrhythmia, Acquired heart disease, No assigned diagnosis, Patent ductus arteriosus (PDA), Pulmonary stenosis (including sub-valvar supra-valvar) | 0–0.42% | 1 |
|  | Isolated ventricular septal defect (VSD), Mitral valve abnormality (including supra-valvar, sub-valvar) | 1.08–1.12% | 2 |
|  | Miscellaneous congenital primary diagnoses, Fallot / double outlet right ventricle (DORV)-Fallot type | 1.38–1.53% | 3 |
|  | Totally anomalous pulmonary venous connection, Transposition of great arteries (TGA) with intact ventricular septum (IVS), Atrioventricular septal defect | 1.68–1.82% | 4 |
|  | Aortic arch obstruction ± ventricular septal defect, Tricuspid valve abnormality (including Ebstein's) | 1.88–1.98% | 5 |
|  | Aortic valve stenosis (isolated), Functionally univentricular heart non-HLHS | 2.68–3.17% | 6 |
|  | Pulmonary atresia and intact ventricular septum (IVS), Transposition of great arteries + ventricular septal defect / double outlet right ventricle | 3.26–3.44% | 7 |
|  | Hypoplastic left heart syndrome (HLHS), Pulmonary atresia + ventricular septal defect (including Fallot type), Interrupted aortic arch, Common arterial trunk (truncus arteriosus) | 3.6–5.34% | 8 |
| Prolonged pleural effusion | Pulmonary atresia and intact ventricular septum (IVS), Subaortic stenosis, Aortic regurgitation, Interatrial communication (ASD), Arrhythmia, Acquired heart disease, No assigned diagnosis, Isolated ventricular septal defect (VSD), Patent ductus arteriosus (PDA), Aortic valve stenosis (isolated) | 0–0.22% | 1 |
|  | Tricuspid valve abnormality (including Ebstein's), Aortic arch obstruction ± ventricular septal defect/ASD, Pulmonary stenosis (including sub-valvar supra-valvar), Transposition of great arteries (concordant AV & discordant VA connections) & IVS, Miscellaneous congenital terms, Totally anomalous pulmonary venous connection, Mitral valve abnormality (including supra-valvar, sub-valvar) | 0.32–0.56% | 2 |
|  | Atrioventricular septal defect, Common arterial trunk (truncus arteriosus), Miscellaneous congenital primary diagnoses | 1.24–1.38% | 3 |
|  | Pulmonary atresia + ventricular septal defect (including Fallot type), Vascular ring, Fallot/DORV-Fallot type | 1.48–1.61% | 4 |
|  | Interrupted aortic arch, Transposition of great arteries + ventricular septal defect/double outlet right ventricle | 2.59–3.41% | 5 |
|  | Functionally univentricular heart – non HLHS, Hypoplastic left heart syndrome (HLHS) | 3.98–4.81% | 6 |
| Acute neurological event | No assigned diagnosis, Subaortic stenosis, Pulmonary stenosis (including sub-valvar supra-valvar) | 0–0.35% | 1 |
|  | Vascular ring, Miscellaneous congenital terms | 0.63–0.75% | 2 |
|  | Patent ductus arteriosus (PDA), Fallot / DORV-Fallot type, Isolated ventricular septal defect (VSD), Interatrial communication (ASD), Aortic arch obstruction ± ventricular septal defect/ASD | 1.00–1.21% | 3 |
|  | Aortic valve stenosis (isolated), Atrioventricular septal defect | 1.83–1.88% | 4 |
|  | Aortic regurgitation, Tricuspid valve abnormality (including Ebstein's), Pulmonary atresia + ventricular septal defect (including Fallot type) | 1.98–2.27% | 5 |
|  | Mitral valve abnormality (including supra-valvar, sub-valvar), Arrhythmia, Transposition of great arteries (concordant AV & discordant VA connections) & IVS, Pulmonary atresia and intact ventricular septum (IVS), Acquired heart disease | 2.82–3.34% | 6 |
|  | Miscellaneous congenital primary diagnoses, Transposition of great arteries + ventricular septal defect/double outlet right ventricle, Common arterial trunk (truncus arteriosus), Functionally univentricular heart – non HLHS | 3.74–4.45% | 7 |
|  | Hypoplastic left heart syndrome (HLHS), Totally anomalous pulmonary venous connection, Interrupted aortic arch | 4.54–6.45% | 8 |
| Extracorporeal life support | Subaortic stenosis, Vascular ring, No assigned diagnosis, Interatrial communication (ASD) | 0–0.15% | 1 |
|  | Patent ductus arteriosus (PDA), Isolated ventricular septal defect (VSD), Aortic arch obstruction ± ventricular septal defect/ASD, Pulmonary stenosis (including sub-valvar supra-valvar), Aortic regurgitation, Totally anomalous pulmonary venous connection | 0.18–0.93% | 2 |
|  | Miscellaneous congenital terms, Atrioventricular septal defect, Tricuspid valve abnormality (including Ebstein's), Arrhythmia, Mitral valve abnormality (including supra-valvar, sub-valvar) | 1.01–1.48% | 3 |
|  | Fallot / DORV-Fallot type, Aortic valve stenosis (isolated) | 1.77–1.83% | 4 |
|  | Functionally univentricular heart – non HLHS, Transposition of great arteries (concordant AV & discordant VA connections) & IVS | 2.12–2.20% | 5 |
|  | Pulmonary atresia + ventricular septal defect (including Fallot type), Pulmonary atresia and intact ventricular septum (IVS), Interrupted aortic arch, Miscellaneous congenital primary diagnoses | 3.06–3.88% | 6 |
|  | Transposition of great arteries + ventricular septal defect/double outlet right ventricle | 4.48% | 7 |
|  | Common arterial trunk (truncus arteriosus), Acquired heart disease, Hypoplastic left heart syndrome (HLHS) | 5.05–6.09% | 8 |
| Renal replacement therapy | Subaortic stenosis, Vascular ring, No assigned diagnosis, Interatrial communication (ASD), Patent ductus arteriosus (PDA), Isolated ventricular septal defect (VSD) | 0–0.39% | 1 |
|  | Miscellaneous congenital terms, Pulmonary stenosis (including sub-valvar supra-valvar), Pulmonary atresia and intact ventricular septum (IVS), Aortic regurgitation, Aortic valve stenosis (isolated) | 0.5–1.38% | 2 |
|  | Mitral valve abnormality (including supra-valvar, sub-valvar), Tricuspid valve abnormality (including Ebstein's), Arrhythmia | 1.63–1.89% | 3 |
|  | Atrioventricular septal defect | 2.24% | 4 |
|  | Aortic arch obstruction ± ventricular septal defect/ASD, Functionally univentricular heart – non HLHS | 2.82–3.06% | 5 |
|  | Fallot / DORV-Fallot type, Miscellaneous congenital primary diagnoses, Pulmonary atresia + ventricular septal defect (including Fallot type) | 3.3–4.29% | 6 |
|  | Transposition of great arteries (concordant AV & discordant VA connections) & IVS, Acquired heart disease, Interrupted aortic arch, Totally anomalous pulmonary venous connection | 5.59–8.39% | 7 |
|  | Common arterial trunk (truncus arteriosus), Transposition of great arteries + ventricular septal defect / double outlet right ventricle, Hypoplastic left heart syndrome (HLHS) | 9.13–9.99% | 8 |
| Unplanned reintervention | Vascular ring, Patent ductus arteriosus (PDA) | 0.32–0.36% | 1 |
|  | Pulmonary stenosis (including sub-valvar supra-valvar), Interatrial communication (ASD) | 0.87–0.96% | 2 |
|  | Transposition of great arteries (concordant AV & discordant VA connections) & IVS, Aortic regurgitation, Isolated ventricular septal defect (VSD) | 1.8–2.04% | 3 |
|  | No assigned diagnosis, Subaortic stenosis, Fallot / DORV-Fallot type | 2.22–2.74% | 4 |
|  | Aortic arch obstruction ± ventricular septal defect/ASD, Miscellaneous congenital terms, Arrhythmia, Totally anomalous pulmonary venous connection, Aortic valve stenosis (isolated), Mitral valve abnormality (including supra-valvar, sub-valvar) | 2.93–3.86% | 5 |
|  | Pulmonary atresia and intact ventricular septum (IVS), Miscellaneous congenital primary diagnoses, Atrioventricular septal defect, Tricuspid valve abnormality (including Ebstein's), Acquired heart disease, Pulmonary atresia + ventricular septal defect (including Fallot type) | 4.97–6.56% | 6 |
|  | Transposition of great arteries + ventricular septal defect/double outlet right ventricle, Functionally univentricular heart – non HLHS | 7.01–7.44% | 7 |
|  | Common arterial trunk (truncus arteriosus), Interrupted aortic arch, Hypoplastic left heart syndrome (HLHS) | 7.93–10.4% | 8 |

## Supplementary Table 4: Specific cardiac procedures as defined and grouped by NCHDA, ranked by prevalence for each complication and the Specific cardiac procedure groups used for complication risk prediction model

[Supporting dataset documentation - NICOR](https://www.nicor.org.uk/datasets/supporting-data-set-documentation)

#### Changes made based on clinical consensus:

For a small number of specific procedures with zero complication outcome events in the sample, the specific procedure was moved to a higher complexity level by clinical consensus and considering related literature indicating the true event number in a larger sample is non-zero. All relevant procedures are marked in the table below.

|  | **Cardiac specific procedure name** | **Prevalence range (%)** | **Complication prevalence group** |
| --- | --- | --- | --- |
| Necrotising enterocolitis | No qualifying cardiac surgery codes in record, Heart transplant, Congenitally corrected transposition of the great arteries repair, Senning or Mustard procedure, Fontan operation, Atrioventricular septal defect (partial) repair, Mitral valve replacement, Ross-Konno procedure, Aortic valve replacement: Ross, Aortic root replacement (not Ross), Aortic valve replacement (non Ross), Tricuspid valve replacement, Pulmonary valve replacement, Unifocalisation procedure (with/without shunt), Tetralogy with absent pulmonary valve repair, VSD with right ventricular outflow tract obstruction (RVOTO) repair, Aortopulmonary window repair, Cor triatriatum repair, Multiple ventricular septal defect (VSD) Closure, Atrial septal defect (ASD) repair, Implantable cardioverter defibrillator (ICD) surgical, Pacemaker lead, Miscellaneous electro physiology (EP) procedures, Vascular ring procedure, Glenn, PDA ligation (surgical) | 0–0.4 | 1 |
|  | VSD Repair, Atrioventricular septal defect (complete) repair | 0.5–0.8 | 2 |
|  | Truncus and interruption repair (move clinician consensus), Arterial switch and VSD closure, Pulmonary atresia VSD repair, Mitral valve repair | 0–1.1 | 3 |
|  | Tetralogy of Fallot-type DORV repair, Pacemaker epicardial, Sub-valvar aortic stenosis repair | 1.3 | 4 |
|  | Isolated coarctation/ hypoplastic aortic arch repair, Arterial switch (complex), Totally anomalous pulmonary venous connection repair | 1.6–1.8 | 5 |
|  | Arterial switch (for isolated transposition), Supra-valvar aortic valve stenosis repair, Aortic valve repair | 2.5–2.7 | 6 |
|  | Atrioventricular septal defect and tetralogy repair, Isolated Pulmonary artery band, Tricuspid valve repair, Arterial switch and aortic arch obstruction repair | 2.8–3.5 | 7 |
|  | Rastelli-REV procedure, Unallocated cardiac surgical procedure, Arterial shunt, Sinus Venosus ASD and-or partial anomalous pulmonary venous connection (PAPVC) repair | 3.6–3.8 | 8 |
|  | Pulmonary vein stenosis procedure, Anomalous coronary artery repair, Interrupted aortic arch repair, Norwood procedure (stage 1) | 4.3–5.8 | 9 |
|  | Truncus arteriosus repair, Right ventricle (RV) to pulmonary artery (PA) conduit construction, Cardiac conduit replacement, Totally anomalous pulmonary venous connection repair and arterial shunt, Pacemaker biventricular | 6.9–25.0 | 10 |
| Prolonged pleural effusion | No qualifying cardiac surgery codes in record, Heart transplant, Truncus and interruption repair, Senning or Mustard procedure, Mitral valve replacement, Ross-Konno procedure, Aortic valve replacement: Ross, Tricuspid valve replacement, Pulmonary valve replacement, Aortic valve repair, Unifocalisation procedure (with/without shunt), Tetralogy with absent pulmonary valve repair, RV to PA conduit construction, VSD RVOTO, Supra-valvar aortic valve stenosis repair, Sub-valvar aortic stenosis repair, Aortopulmonary window repair, Anomalous coronary artery repair, Cor triatriatum repair, Cardiac conduit replacement, VSD Repair, Sinus Venosus ASD and-or PAPVC repair, ASD repair, PDA ligation (surgical), Arrhythmia Surgical, Pacemaker epicardial, ICD surgical, Pacemaker biventricular, Pacemaker lead, Miscellaneous EP procedures, Isolated coarctation/ hypoplastic aortic arch repair, Tricuspid valve repair | 0–0.3 | 1 |
|  | Unallocated cardiac surgical procedure, Atrioventricular septal defect (partial) repair, Mitral valve repair | 0.4–0.5 | 2 |
|  | Totally anomalous pulmonary venous connection repair and arterial shunt, Norwood procedure (stage 1), Isolated Pulmonary artery band, Arterial shunt, Totally anomalous pulmonary venous connection repair | 0–1.0 | 3 |
|  | Vascular ring procedure, Aortic valve replacement (non Ross), Glenn, Truncus arteriosus repair | 1.1–1.3 | 4 |
|  | Arterial switch (for isolated transposition), Atrioventricular septal defect (complete) repair, Pulmonary atresia VSD repair | 1.5–1.9 | 5 |
|  | Pulmonary vein stenosis procedure, Tetralogy of Fallot-type DORV repair, Multiple VSD Closure | 2.1–2.9 | 6 |
|  | Atrioventricular septal defect and tetralogy repair, Arterial switch and VSD closure, Aortic root replacement (not Ross), Interrupted aortic arch repair, Arterial switch (complex), Arterial switch and aortic arch obstruction repair | 3.1–4.5 | 7 |
|  | Congenitally corrected transposition of the great arteries repair, Rastelli-REV procedure, Fontan | 10.0–13.8 | 8 |
| Acute neurological event | No qualifying cardiac surgery codes in record, Multiple VSD Closure, Arrhythmia Surgical, EP Miscellaneous, Pacemaker epicardial, VSD Repair | 0–0.5 | 1 |
|  | Vascular ring procedure, Sub-valvar aortic stenosis repair, ASD repair, Tetralogy of Fallot-type DORV repair, PDA ligation (surgical) | 0.7–0.9 | 2 |
|  | Truncus and interruption repair (clinician consensus), Senning or Mustard procedure (clinician consensus), Tricuspid valve replacement (clinician consensus), Atrioventricular septal defect (partial) repair, Aortic valve repair, Isolated coarctation/ hypoplastic aortic arch repair, Pulmonary valve replacement | 0–1.3 | 3 |
|  | Aortopulmonary window repair, Atrioventricular septal defect (complete) repair, Cor triatriatum repair | 1.5–1.8 | 4 |
|  | Sinus Venosus ASD and-or PAPVC repair, RV to PA conduit construction, Fontan, Cardiac conduit replacement, Mitral valve repair, Tricuspid valve repair | 2.0–2.3 | 5 |
|  | Aortic root replacement (not Ross), Unifocalisation procedure (with/without shunt), Anomalous coronary artery repair, Arterial switch (for isolated transposition), Pulmonary atresia VSD repair, VSD RVOTO, Pacemaker lead, Arterial switch and VSD closure, Unallocated cardiac surgical procedure | 2.4–3.0 | 6 |
|  | Ross-Konno procedure, Aortic valve replacement: Ross, Isolated Pulmonary artery band, Arterial shunt, Rastelli-REV procedure, Truncus arteriosus repair | 3.1–3.7 | 7 |
|  | Glenn, Pulmonary vein stenosis procedure, Pacemaker biventricular | 4.0–4.5 | 8 |
|  | Heart transplant, Tetralogy with absent pulmonary valve repair, Aortic valve replacement (non Ross), Mitral valve replacement, Arterial switch (complex), Totally anomalous pulmonary venous connection repair, Supra-valvar aortic valve stenosis repair | 5.0–5.6 | 9 |
|  | Congenitally corrected transposition of the great arteries repair, Atrioventricular septal defect and tetralogy repair, Norwood procedure (stage 1), Interrupted aortic arch repair, ICD surgical, Arterial switch and aortic arch obstruction repair, Totally anomalous pulmonary venous connection repair and arterial shunt | 6.3–28.6 | 10 |
| Extracorporeal life support | No qualifying cardiac surgery codes in record, Multiple VSD Closure, Sinus Venosus ASD and-or PAPVC repair, ASD repair, PDA ligation (surgical), Arrhythmia Surgical, ICD surgical, Pacemaker biventricular, Pacemaker lead, EP Miscellaneous, Vascular ring procedure, VSD Repair, Isolated Pulmonary artery band, Isolated coarctation/ hypoplastic aortic arch repair, Pacemaker epicardial | 0–0.2 | 1 |
|  | Senning or Mustard procedure, (clinician consensus), Pulmonary vein stenosis procedure, (clinician consensus), Subvalvar aortic stenosis repair, Pulmonary valve replacement, Atrioventricular septal defect (partial) repair, VSD RVOTO, Fontan | 0–0.9 | 2 |
|  | Atrioventricular septal defect (complete) repair, Glenn, Cardiac conduit replacement, Mitral valve repair | 1.1–1.4 | 3 |
|  | Totally anomalous pulmonary venous connection repair, Aortic valve repair, Tricuspid valve repair, Pulmonary atresia VSD repair | 1.5–1.7 | 4 |
|  | Cor triatriatum repair, RV to PA conduit construction, Tetralogy of Fallot-type DORV repair | 1.8–1.9 | 5 |
|  | Aortic valve replacement: Ross, Unallocated cardiac surgical procedure | 2.2 | 6 |
|  | Tetralogy with absent pulmonary valve repair, Tricuspid valve replacement, Unifocalisation procedure (with/without shunt), Arterial switch (for isolated transposition), Supra-valvar aortic valve stenosis repair | 2.5–2.8 | 7 |
|  | Aortopulmonary window repair, Aortic valve replacement (non Ross), Ross-Konno procedure, Aortic root replacement (not Ross), Atrioventricular septal defect and tetralogy repair, Rastelli-REV procedure | 3.1–4.8 | 8 |
|  | Mitral valve replacement, Interrupted aortic arch repair, Arterial switch and VSD closure, Arterial switch and aortic arch obstruction repair, Arterial switch (complex), Truncus arteriosus repair, Arterial shunt, Anomalous coronary artery repair | 5.6–9.9 | 9 |
|  | Congenitally corrected transposition of the great arteries repair, Norwood procedure (stage 1), Totally anomalous pulmonary venous connection repair and arterial shunt, Heart transplant, Truncus and interruption repair | 12.4–22.2 | 10 |
| Renal replacement therapy | No qualifying cardiac surgery codes in record, Tricuspid valve replacement, Aortopulmonary window repair, Multiple VSD Closure, Sinus Venosus ASD and-or PAPVC repair, ASD repair, Arrhythmia Surgical, ICD surgical, Pacemaker biventricular, Pacemaker lead, EP Miscellaneous, PDA ligation (surgical), VSD Repair, Vascular ring procedure | 0–0.1 | 1 |
|  | Pacemaker epicardial, Aortic valve replacement: Ross, Subvalvar aortic stenosis repair, Glenn | 0.2–0.6 | 2 |
|  | Totally anomalous pulmonary venous connection repair and arterial shunt, (clinician consensus), Mitral valve repair, Pulmonary valve replacement, Isolated Pulmonary artery band, Aortic valve repair, Supra-valvar aortic valve stenosis repair | 0–0.9 | 3 |
|  | Pulmonary vein stenosis procedure, Atrioventricular septal defect (partial) repair, RV to PA conduit construction, Aortic root replacement (not Ross), Tricuspid valve repair, Cor triatriatum repair, Isolated coarctation/ hypoplastic aortic arch repair, Cardiac conduit replacement, Fontan | 1.0–1.9 | 4 |
|  | VSD RVOTO, Atrioventricular septal defect (complete) repair, Arterial shunt, Mitral valve replacement, Unallocated cardiac surgical procedure, Rastelli-REV procedure, Aortic valve replacement (non Ross) | 2.0–3.8 | 5 |
|  | Tetralogy of Fallot-type DORV repair, Unifocalisation procedure (with/without shunt), Ross-Konno procedure | 4.1–5.2 | 6 |
|  | Anomalous coronary artery repair, Tetralogy with absent pulmonary valve repair, Arterial switch (for isolated transposition) | 7.2–7.8 | 7 |
|  | Congenitally corrected transposition of the great arteries repair, Atrioventricular septal defect and tetralogy repair, Pulmonary atresia VSD repair, Totally anomalous pulmonary venous connection repair, Interrupted aortic arch repair | 8.6–12.3 | 8 |
|  | Arterial switch and VSD closure, Arterial switch (complex), Heart transplant, Arterial switch and aortic arch obstruction repair | 16.1–20.2 | 9 |
|  | Truncus arteriosus repair, Truncus and interruption repair, Senning or Mustard procedure, Norwood procedure (stage 1) | 21.2–23.9 | 10 |
| Unplanned reintervention | No qualifying cardiac surgery codes in record, Senning or Mustard procedure, Arrhythmia Surgical, Pacemaker biventricular, ASD repair | 0–0.7 | 1 |
|  | PDA ligation (surgical), Sinus Venosus ASD and-or PAPVC repair, Pulmonary valve replacement, Vascular ring procedure, Pacemaker epicardial | 1.0–1.6 | 2 |
|  | Aortopulmonary window repair, Cardiac conduit replacement, VSD Repair | 1.5–1.9 | 3 |
|  | Isolated coarctation/ hypoplastic aortic arch repair, Atrioventricular septal defect (partial) repair, VSD RVOTO | 2.1–2.4 | 4 |
|  | Aortic valve replacement: Ross, Tetralogy of Fallot-type DORV repair | 2.7–2.9 | 5 |
|  | Pulmonary vein stenosis procedure, Arterial switch (for isolated transposition), RV to PA conduit construction, Multiple VSD Closure, Cor triatriatum repair, Anomalous coronary artery repair, Supra-valvar aortic valve stenosis repair | 3.1–3.7 | 6 |
|  | Totally anomalous pulmonary venous connection repair and arterial shunt, (moved clinician consensus), Aortic valve repair, Isolated Pulmonary artery band, Subvalvar aortic stenosis repair, Aortic root replacement (not Ross), ICD surgical, Arterial switch and VSD closure | 0–5.0 | 7 |
|  | Unallocated cardiac surgical procedure, Aortic valve replacement (non Ross), Tricuspid valve repair, Mitral valve repair, Ross-Konno procedure, Atrioventricular septal defect (complete) repair, Glenn, Totally anomalous pulmonary venous connection repair | 5.1–5.8 | 8 |
|  | Fontan, Miscellaneous electrophysiology operations, Arterial switch and aortic arch obstruction repair, Tetralogy with absent pulmonary valve repair, Interrupted aortic arch repair, Pacemaker lead, Heart transplant, Pulmonary atresia VSD repair, Arterial switch (complex), Atrioventricular septal defect and tetralogy repair | 5.9–10.3 | 9 |
|  | Arterial shunt, Truncus and interruption repair, Truncus arteriosus repair, Unifocalisation procedure (with/without shunt), Norwood procedure (stage 1), Mitral valve replacement, Rastelli-REV procedure, Tricuspid valve replacement, Congenitally corrected transposition of the great arteries repair | 10.5–27.8 | 10 |

## Supplementary Table 5: Variables included in each complication model

These risk factor variables were included in the final multi-variable risk model for the stated complication outcome based on our prior study

*Risk models for monitoring post-operative complication rates after pediatric cardiac surgery authors - Hannah K Mitchell, Ferran Espuny Pujol, Rodney C. Franklin, Gareth Ambler, John Stickley, Julie A. Taylor, Carin Van Doorn, Serban Stoica, Victor Tsang, Christina Pagel, Sonya Crowe, Katherine L. Brown*

| **Complication** | **Variables selected for final model** |
| --- | --- |
| Acute neurological event | procedure urgency, acquired comorbidity, congenital non cardiac comorbidity, severity of illness, presence of functionally univentricular heart, procedure group and diagnostic group |
| Unplanned re-operation | age, weight, procedure urgency, acquired comorbidity, additional cardiac risk factors, congenital non cardiac comorbidity, congenital cardiac risk factors, severity of illness, procedure group and diagnostic group |
| Renal replacement therapy | age, age square root, weight square root, procedure urgency, acquired comorbidity, severity of illness, procedure group and diagnostic group |
| Extracorporeal life support | sex, weight, weight square root, procedure urgency, additional cardiac risk factors, congenital non cardiac comorbidity, severity of illness, procedure group and diagnostic group |
| Necrotising enterocolitis | age square root, weight square root, acquired comorbidity, congenital non cardiac comorbidity, prematurity, presence of functionally univentricular heart, procedure group and diagnostic group |
| Prolonged pleural effusion | sex, age square root, weight, congenital non cardiac comorbidity, presence of functionally univentricular heart, procedure group and diagnostic group |

## Supplementary Table 6a: Baseline characteristics of cohort by ethnic group (n= 23,423)

|  |  | Total | Asian | Black | Mixed | Other | White | Unknown | p-value |
| --- | --- | --- | --- | --- | --- | --- | --- | --- | --- |
|  |  | N=23,423 | N=2,678 (11.4%) | N=997 (4.3%) | N=531 (2.3%) | N=977 (4.2%) | N=14,537 (62.1%) | N=3,703 (15.8%) | |
| Weight (kg) median IQR | | 7.0 (3.9-15.1) | 7.4 (4.1-15.4) | 7.1 (4.0-15.6) | 6.6 (3.9-12.9) | 6.8 (3.8-15.0) | 7.2 (4.0-15.8) | 6.0 (3.5-12.2) | <0.001 |
| Age (Years) median IQR | | 0.6 (0.2-4.0) | 0.7 (0.2-4.4) | 0.6 (0.2-4.0) | 0.6 (0.2-2.8) | 0.6 (0.2-4.0) | 0.7 (0.2-4.2) | 0.5 (0.1-2.4) | <0.001 |
| Sex | Male | 13,013 (55.6%) | 1,496 (55.9%) | 554 (55.6%) | 283 (53.3%) | 562 (57.5%) | 8,049 (55.4%) | 2,069 (55.9%) | 0.92 |
|  | Female | 10,405 (44.4%) | 1,182 (44.1%) | 443 (44.4%) | 248 (46.7%) | 415 (42.5%) | 6,484 (44.6%) | 1,633 (44.1%) |  |
| Financial Year | 2015 | 3,751 (16.0%) | 429 (16.0%) | 185 (18.6%) | 0 (0.0%) | 189 (19.3%) | 2,463 (16.9%) | 485 (13.1%) | <0.001 |
|  | 2016 | 3,721 (15.9%) | 406 (15.2%) | 193 (19.4%) | 3 (0.6%) | 190 (19.4%) | 2,394 (16.5%) | 535 (14.4%) |  |
|  | 2017 | 3,543 (15.1%) | 392 (14.6%) | 137 (13.7%) | 124 (23.4%) | 125 (12.8%) | 2,315 (15.9%) | 450 (12.2%) |  |
|  | 2018 | 3,542 (15.1%) | 345 (12.9%) | 139 (13.9%) | 80 (15.1%) | 122 (12.5%) | 1,927 (13.3%) | 929 (25.1%) |  |
|  | 2019 | 3,268 (14.0%) | 421 (15.7%) | 115 (11.5%) | 115 (21.7%) | 88 (9.0%) | 1,938 (13.3%) | 591 (16.0%) |  |
|  | 2020 | 2,832 (12.1%) | 330 (12.3%) | 103 (10.3%) | 106 (20.0%) | 133 (13.6%) | 1,767 (12.2%) | 393 (10.6%) |  |
|  | 2021 | 2,766 (11.8%) | 355 (13.3%) | 125 (12.5%) | 103 (19.4%) | 130 (13.3%) | 1,733 (11.9%) | 320 (8.6%) |  |
| IMD quintile | 1 (less deprived) | 2,971 (12.7%) | 159 (5.9%) | 42 (4.2%) | 71 (13.4%) | 68 (7.0%) | 2,214 (15.2%) | 417 (11.3%) | <0.001 |
|  | 2 | 3,240 (13.8%) | 206 (7.7%) | 58 (5.8%) | 84 (15.8%) | 100 (10.2%) | 2,324 (16.0%) | 468 (12.6%) |  |
|  | 3 | 3,867 (16.5%) | 379 (14.2%) | 135 (13.5%) | 87 (16.4%) | 149 (15.3%) | 2,587 (17.8%) | 530 (14.3%) |  |
|  | 4 | 4,601 (19.6%) | 668 (24.9%) | 273 (27.4%) | 95 (17.9%) | 175 (17.9%) | 2,621 (18.0%) | 769 (20.8%) |  |
|  | 5 (more deprived) | 6,143 (26.2%) | 1,115 (41.6%) | 435 (43.6%) | 158 (29.8%) | 278 (28.5%) | 3,183 (21.9%) | 974 (26.3%) |  |
|  | Missing | 2,601 (11.1%) | 151 (5.6%) | 54 (5.4%) | 36 (6.8%) | 207 (21.2%) | 1,608 (11.1%) | 545 (14.7%) |  |
| Acquired Comorbidity | | 3,559 (15.2%) | 422 (15.8%) | 162 (16.2%) | 78 (14.7%) | 149 (15.3%) | 2,197 (15.1%) | 551 (14.9%) | 0.85 |
| Additional Cardiac Risk Factors | | 1,747 (7.5%) | 187 (7.0%) | 70 (7.0%) | 42 (7.9%) | 79 (8.1%) | 1,084 (7.5%) | 285 (7.7%) | 0.82 |
| Congenital Comorbidity | | 4,875 (20.8%) | 548 (20.5%) | 242 (24.3%) | 124 (23.4%) | 192 (19.7%) | 3,054 (21.0%) | 715 (19.3%) | 0.008 |
| Congenital Cardiac Risk Factors | | 370 (1.6%) | 85 (3.2%) | 15 (1.5%) | 3 (0.6%) | 14 (1.4%) | 215 (1.5%) | 38 (1.0%) | <0.001 |
| Down Syndrome | | 1,842 (7.9%) | 175 (6.5%) | 153 (15.3%) | 47 (8.9%) | 87 (8.9%) | 1,033 (7.1%) | 347 (9.4%) | <0.001 |
| Premature | | 3,144 (13.4%) | 309 (11.5%) | 153 (15.3%) | 68 (12.8%) | 124 (12.7%) | 1,876 (12.9%) | 614 (16.6%) | <0.001 |
| Severity of illness | | 3,281 (14.0%) | 350 (13.1%) | 137 (13.7%) | 57 (10.7%) | 124 (12.7%) | 1,965 (13.5%) | 648 (17.5%) | <0.001 |
| Functionally univentricular heart | | 3,333 (14.2%) | 455 (17.0%) | 140 (14.0%) | 74 (13.9%) | 143 (14.6%) | 2,206 (15.2%) | 315 (8.5%) | <0.001 |
| Elective y/n | Elective | 15,904 (67.9%) | 1,871 (69.9%) | 669 (67.1%) | 369 (69.5%) | 688 (70.4%) | 9,858 (67.8%) | 2,449 (66.1%) | 0.044 |
|  | Urgent, emergency, salvage | 7,430 (31.7%) | 794 (29.6%) | 324 (32.5%) | 162 (30.5%) | 286 (29.3%) | 4,629 (31.8%) | 1,235 (33.4%) |  |

## Supplementary Table 6b: Baseline characteristics of cohort by fifth of deprivation (index of multiple deprivation) (n= 23,423)

|  |  | 1 (less deprived) | 2 | 3 | 4 | 5 (more deprived) | Missing | P value |
| --- | --- | --- | --- | --- | --- | --- | --- | --- |
|  |  | N=2,971 (12.7%) | N=3,240 (13.8%) | N=3,867 (16.5%) | N=4,601 (19.6%) | N=6,143 (26.2%) | N=2,601 (11.1%) | |
| Weight (kg) median IQR | | 7.5 (4.1-16.6) | 7.0 (3.8-15.4) | 6.8 (3.8-15.0) | 6.6 (3.7-14.2) | 7.0 (3.9-14.9) | 7.4 (4.2-15.9) | <0.001 |
| Age (Years) median IQR | | 0.7 (0.2-4.5) | 0.6 (0.2-4.0) | 0.6 (0.2-3.8) | 0.6 (0.2-3.5) | 0.6 (0.2-3.9) | 0.7 (0.2-4.5) | <0.001 |
| Sex | Male | 1,644 (55.3%) | 1,793 (55.3%) | 2,174 (56.2%) | 2,533 (55.1%) | 3,421 (55.7%) | 1,448 (55.7%) | 0.73 |
|  | Female | 1,327 (44.7%) | 1,445 (44.6%) | 1,693 (43.8%) | 2,066 (44.9%) | 2,721 (44.3%) | 1,153 (44.3%) |  |
| Financial Year | 2015 | 490 (16.5%) | 522 (16.1%) | 606 (15.7%) | 759 (16.5%) | 937 (15.3%) | 437 (16.8%) | 0.004 |
|  | 2016 | 451 (15.2%) | 502 (15.5%) | 611 (15.8%) | 706 (15.3%) | 991 (16.1%) | 460 (17.7%) |  |
|  | 2017 | 446 (15.0%) | 482 (14.9%) | 591 (15.3%) | 645 (14.0%) | 951 (15.5%) | 428 (16.5%) |  |
|  | 2018 | 444 (14.9%) | 522 (16.1%) | 588 (15.2%) | 686 (14.9%) | 926 (15.1%) | 376 (14.5%) |  |
|  | 2019 | 427 (14.4%) | 433 (13.4%) | 567 (14.7%) | 674 (14.6%) | 877 (14.3%) | 290 (11.1%) |  |
|  | 2020 | 346 (11.6%) | 389 (12.0%) | 483 (12.5%) | 602 (13.1%) | 724 (11.8%) | 288 (11.1%) |  |
|  | 2021 | 367 (12.4%) | 390 (12.0%) | 421 (10.9%) | 529 (11.5%) | 737 (12.0%) | 322 (12.4%) |  |
| Ethnicity | Asian | 2,214 (74.5%) | 2,324 (71.7%) | 2,587 (66.9%) | 2,621 (57.0%) | 3,183 (51.8%) | 1,608 (61.8%) | <0.001 |
|  | Black | 71 (2.4%) | 84 (2.6%) | 87 (2.2%) | 95 (2.1%) | 158 (2.6%) | 36 (1.4%) |  |
|  | Mixed | 159 (5.4%) | 206 (6.4%) | 379 (9.8%) | 668 (14.5%) | 1,115 (18.2%) | 151 (5.8%) |  |
|  | Other | 42 (1.4%) | 58 (1.8%) | 135 (3.5%) | 273 (5.9%) | 435 (7.1%) | 54 (2.1%) |  |
|  | White | 68 (2.3%) | 100 (3.1%) | 149 (3.9%) | 175 (3.8%) | 278 (4.5%) | 207 (8.0%) |  |
|  | Unknown | 417 (14.0%) | 468 (14.4%) | 530 (13.7%) | 769 (16.7%) | 974 (15.9%) | 545 (21.0%) |  |
| Acquired Comorbidity | | 379 (12.8%) | 455 (14.0%) | 533 (13.8%) | 726 (15.8%) | 1,052 (17.1%) | 414 (15.9%) | <0.001 |
| Additional Cardiac Risk Factors | | 199 (6.7%) | 225 (6.9%) | 268 (6.9%) | 346 (7.5%) | 506 (8.2%) | 203 (7.8%) | 0.048 |
| Congenital Comorbidity | | 577 (19.4%) | 618 (19.1%) | 781 (20.2%) | 949 (20.6%) | 1,359 (22.1%) | 591 (22.7%) | <0.001 |
| Congenital Cardiac Risk Factors | | 24 (0.8%) | 33 (1.0%) | 62 (1.6%) | 80 (1.7%) | 108 (1.8%) | 63 (2.4%) | <0.001 |
| Down Syndrome | | 213 (7.2%) | 239 (7.4%) | 315 (8.1%) | 344 (7.5%) | 517 (8.4%) | 214 (8.2%) | 0.19 |
| Premature | | 374 (12.6%) | 450 (13.9%) | 480 (12.4%) | 665 (14.5%) | 873 (14.2%) | 302 (11.6%) | <0.001 |
| Severity of illness | | 358 (12.0%) | 427 (13.2%) | 505 (13.1%) | 684 (14.9%) | 961 (15.6%) | 346 (13.3%) | <0.001 |
| Functionally univentricular heart | | 293 (9.9%) | 373 (11.5%) | 500 (12.9%) | 655 (14.2%) | 960 (15.6%) | 552 (21.2%) | <0.001 |
| Elective y/n | Elective | 2,081 (70.0%) | 2,186 (67.5%) | 2,646 (68.4%) | 3,093 (67.2%) | 4,073 (66.3%) | 1,825 (70.2%) | 0.001 |
|  | Urgent, emergency, salvage | 876 (29.5%) | 1,042 (32.2%) | 1,207 (31.2%) | 1,483 (32.2%) | 2,050 (33.4%) | 772 (29.7%) |  |

Supplementary Table 7: Sensitivity analysis—univariable and adjusted associations between ethnicity, deprivation, and complication occurrence (multiple imputation for missing ethnicity)

|  | ANE | | | | URO | | | | Renal replacement therapy | | |  |
| --- | --- | --- | --- | --- | --- | --- | --- | --- | --- | --- | --- | --- |
|  | OR (95% CI) | p-value | aOR (95% CI) | p-value | OR (95% CI) | p-value | aOR (95% CI) | p-value | OR (95% CI) | p-value | aOR (95% CI) | p-value |
| White | Ref |  | Ref | 0.4 | Ref |  | Ref | 0.4 | Ref |  | Ref | **0.02** |
| Asian | 1.27 (0.90–1.80) | 0.173 | 1.23 (0.88–1.73) |  | 0.89 (0.66–1.19) | 0.42 | 0.85 (0.64–1.14) |  | 0.91 (0.70–1.18) | 0.465 | 0.91 (0.69–1.21) |  |
| Black | 1.00 (0.57–1.77) | 0.988 | 1.05 (0.58–1.90) |  | 0.89 (0.60–1.31) | 0.542 | 0.89 (0.61–1.29) |  | 0.97 (0.61–1.54) | 0.904 | 1.05 (0.67–1.65) |  |
| Mixed | 0.62 (0.34–1.14) | 0.121 | 0.65 (0.36–1.16) |  | 1.01 (0.66–1.54) | 0.975 | 1.06 (0.68–1.64) |  | 1.12 (0.72–1.75) | 0.601 | 1.20 (0.78–1.86) |  |
| Other | 1.11 (0.62–1.98) | 0.723 | 1.15 (0.69–2.17) |  | 0.73 (0.53–1.00) | **0.049** | 0.75 (0.55–1.02) |  | 0.60 (0.40–0.90) | **0.013** | 0.59 (0.40–0.87) |  |
|  |  |  |  |  |  |  |  |  |  |  |  |  |
|  | Extracorporeal life support | | | | Necrotising enterocolitis | | | | Prolonged pleural effusion | | | |
|  | OR (95% CI) | p-value | aOR (95% CI) | p-value | OR (95% CI) | p-value | aOR (95% CI) | p-value | OR (95% CI) | p-value | aOR (95% CI) | p-value |
| White | Ref |  | Ref | **0.02** | Ref |  | Ref | **0.03** | Ref |  | Ref | **0.02** |
| Asian | 0.99 (0.73–1.34) | 0.956 | 0.99 (0.70–1.40) |  | 1.22 (0.85–1.75) | 0.27 | 1.08 (0.74–1.58) |  | 2.27 (1.14–4.52) | **0.019** | 1.78 (0.96–3.30) |  |
| Black | 1.03 (0.65–1.64) | 0.885 | 1.16 (0.77–1.74) |  | 1.01 (0.43–2.39) | 0.984 | 1.12 (0.47–2.65) |  | 0.76 (0.43–1.32) | 0.326 | 0.68 (0.38–1.23) |  |
| Mixed | 0.36 (0.15–0.85) | **0.019** | 0.39 (0.16–0.94) |  | 1.16 (0.42–3.19) | 0.771 | 1.19 (0.44–3.24) |  | 1.55 (0.97–2.46) | 0.065 | 1.39 (0.87–2.22) |  |
| Other | 0.64 (0.40–1.04) | 0.074 | 0.69 (0.42–1.15) |  | 1.92 (1.31–2.82) | **0.001** | 1.82 (1.20–2.75) |  | 0.30 (0.11–0.86) | **0.024** | 0.31 (0.10–0.98) |  |

1. The European Paediatric Cardiac Code (EPCC) is part of the International Paediatric and Congenital Cardiac Code (IPCCC), a standardised terminology used for recording diagnoses, procedures, and complications in paediatric and congenital cardiac care. [↑](#footnote-ref-1)
